# The effect of motor interference therapy in traumatic memories: A pilot study

**DOI:** 10.1002/brb3.1984

**Published:** 2020-12-13

**Authors:** Alonso Morales‐Rivero, Lorena Reyes‐Santos, Erik Bisanz, Angel Ruiz‐Chow, Daniel Crail‐Melendez

**Affiliations:** ^1^ Neuropsychiatry Unit National Institute of Neurology and Neurosurgery Mexico City Mexico; ^2^ University of British Columbia Vancouver BC Canada; ^3^ Faculty of Medicine UNIVERSIDAD NACIONAL AUTONOMA DE MEXICO Mexico City Mexico

**Keywords:** motor interference, post‐traumatic stress disorder, stress‐related disorders, traumatic memories

## Abstract

**Introduction:**

Traumatic memories of events such as a life‐threatening incident, serious injury, or sexual violence are a core symptom of stress‐related disorders; they might be susceptible to positive modification with interference tasks (reconsolidation‐based interventions). Our objective was to test the effect of performing a motor interference task (finger tapping in response to audio cues) on patients who suffer from traumatic memories.

**Methods:**

We designed an uncontrolled pilot prospective clinical trial. Ten participants listened to an audio track that instructed them to tap their fingers in response to specific audio cues while trying to recall the traumatic event. Each patient underwent an assessment including the Spanish version of the PTSD Symptom Severity Scale‐Revised (EGS‐R), the visual analogue scale (EQ‐VAS) from EuroQol 5D (EQ‐5D), and a simple visual analogue scale (VAS) before the intervention, immediately after, and a week after the treatment.

**Results:**

All measures exhibited a statistically significant improvement 1 week after the study. On the PTSD scale, 1 week later, 30% of the patients did not score high enough for such diagnosis. The VAS measured immediately following the intervention (4.4, *SD* = 2.22) also improved (*p *< .001), and 30% of the patients scored zero. One week after the intervention, the VAS improved more than 50%

**Conclusion:**

The rapid 1‐week improvement on the PSTD scale and the VAS after a 30 min intervention support the idea of further research using a double‐blind, controlled design powered to demonstrate the efficacy of motor interference, an easy‐to‐apply therapeutic tool, in the treatment of traumatic memories.

## Graphical abstract

Our objective was to test the effect of performing a motor interference task (finger tapping in response to audio cues) on patients who suffer from traumatic memories. All the patients exhibited a clinical improvement a week after the intervention.

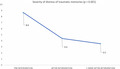

## 1 INTRODUCTION

In order for trauma‐related disorders to develop, an individual must be exposed either directly or indirectly to an extreme stressor (Howlett & Stein, 2016; Jorge, 2015). Direct exposure includes events such as a life‐threatening incident, serious injury, or sexual violence and indirect exposure could happen by occupational exposure or by learning a relative has undergone trauma. Traumatic memories of events are a core symptom of stress‐related disorders and one of the main criteria for diagnosis (American Psychiatric Association, 2014).

Stress‐related symptoms are the result of an increased response to threatening stimuli and an inability of cognitive structures to maintain control over traumatic memories. Implicated regions include the amygdala, hippocampus, and prefrontal cortex (cingulated gyrus, orbitofrontal cortex, ventromedial cortex) (Liberzon & Sripada, 2007; Martin et al., 2009; Rauch et al., 2006). The lack of modulation of the cingulated gyrus (Bremner et al., 2004) and left ventromedial prefrontal cortex over the left amygdala allows for an assignment of exaggerated emotional relevance to the stimuli and the perception of the experience as harmful, unpleasant, or threatening (Rauch et al., 2006). This response to stimuli is retained through the memory consolidation process. When memories are recalled and reactivated, a reconsolidation process occurs, which might be susceptible to positive modification (Sara, 2000) using pharmacological or behavioral strategies (Walsh et al., 2018).

One possible intervention is through retroactive interference that competes with the reconsolidation process (Postman & Underwood, 1973). Verbal and nonverbal cognitive processes are fundamental to the encoding and understanding of traumatic memories and could be modified by the intervention of executive and motor tasks (Brewin, 2007; Holmes et al., 2004). Using this principle, we sought to test a brief and inexpensive treatment for patients with traumatic memories using a motor interference therapy, specifically, finger‐tapping tasks associated with audio stimuli.

## 2 METHODS

We designed an uncontrolled pilot prospective clinical trial. Participants were recruited from Mexico's National Institute of Neurology and Neurosurgery. Inclusion criteria were age (minimum 16 years), Spanish as a native language, and at least one traumatic memory causing distress. All participants provided written, informed consent. We excluded patients with any neurological or psychiatric disorder impacting verbal comprehension or judgment, or with any hearing impairment.

We performed an initial interview applying the Spanish version of the PTSD Symptom Severity Scale‐Revised (EGS‐R) (Echeburúa et al., 2016), the visual analogue scale (EQ‐VAS) from EuroQol 5D (EQ‐5D) (Badia et al., 1999) as a gauge of health‐related quality of life, and a simple visual analogue scale (VAS) in which patients rated the level of distress provoked by their traumatic memories from 0 to 10. All the scales were clinician administered. The participants, who wore headphones, then listened to an audio track twice and followed its instructions. The audio track, designed by Tim Phizackerley, played for 14 min. The evaluation lasted less than 30 min. The first 4 min of the audio track instructed the subjects to tap their fingers in response to specific sounds: Right‐handed tapping with high‐pitched fast sound, left‐handed tapping with high‐pitched slow sound, and bilateral tapping with the low‐pitched continuous sound. During the remaining 10 min, the patients were asked to recall a traumatic memory while simultaneously tapping their fingers. The series of motors tasks included both hands, because the finger tapping responded to specific sounds and directions that appeared in an unpredictable sequence.

Two observers registered the time in which the patient performed the tapping. When the patient stopped tapping, the observer stopped the timer and reinitiated it when the patient reinitiated the task. By comparing the tapping time versus the length of the audio, we assured patients completed at least 80% of the motor task. When they were finished, we readministered the VAS to measure the level of distress caused by a traumatic memory. A week after the study, we reassessed patients using all three scales.

The study protocol was authorized and approved by the Ethics and Investigation Committee of the National Institute of Neurology and Neurosurgery in Mexico City.

We performed a descriptive analysis of dimensional variables with central tendency measures using the SPSS version 17.0 for Windows (SPSS Inc, 2008). The base scores of the scales were compared with the postintervention scores by means of paired sample *t* test.

## 3 RESULTS

Ten patients were included in the study (7 women and 3 men), with a mean age of 30 years. Three patients were excluded (1 with hearing impairment, 1 traumatic head injury, and 1 less than 80% of the motor task completed). They had an average education of 13.9 years. The average months elapsed after exposure to the traumatic event were 147. Seven participants had only one traumatic memory, 3 of them suffered 2 or more. In those patients with 2 or more memories, we asked them to choose one traumatic memory for the intervention. We did not assess the effect of the intervention in the other traumatic memories but only in the chosen one. Anecdotally one of them reported overall improvement. All participants fulfilled the PTSD criteria in the initial assessment, and three suffered from comorbid major depressive disorder and generalized anxiety disorder.

**Figure 1:**
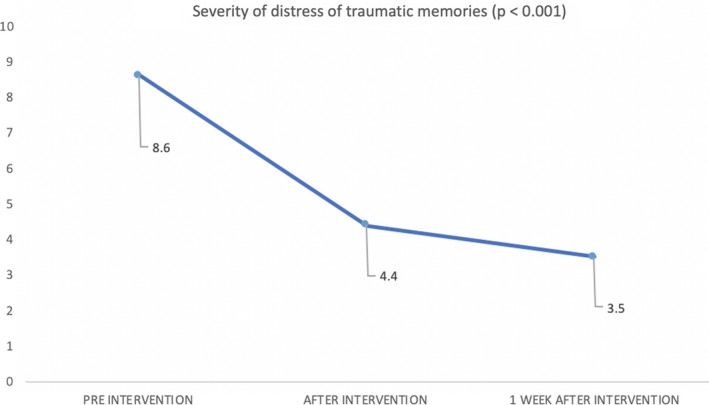
Simple visual analogue scale (VAS) scores showing a statistically significant improvement in the level of distress associated with traumatic memories pre‐, post‐, and 1 week after intervention

Pre‐ and postintervention scores are provided in Table 1. All measures exhibited a statistically significant improvement 1 week after the study. On the (EGS‐R) scale, 100% of the patients had initial scores compatible with PTSD diagnosis, whereas 1 week later 30% of the patients did not score high enough for such diagnosis. We used the (EGS‐R) anxiety, arousal, re‐experience, and avoidance subscales also as outcome measures to asses specific changes on those symptoms. The VAS measured immediately following the intervention (4.4, *SD* = 2.22) also improved (*p *< .001), as shown in Figure 1, and 30% of the patients scored zero. The visual analogue scale (EQ‐VAS) from EuroQol 5D (EQ‐5D) also showed improvement. See Table 1 and Figure 1.

**Table 1:** Pre‐ and postintervention scores

Scale	Preintervention	1 week postintervention	Mean difference	*p*
Total PTSD score	34.10 (*SD* 7.17)	19.80 ( *SD* 11.51)	14.3 (*SD* 9.79)	.001
Re‐experience subscale	10.10 (*SD* 2.23)	5.80 (*SD* 3.45)	4.30 (*SD* 3.86)	.006
Avoidance subscales	12.90 (*SD* 3.81)	7.20 (*SD* 4.44)	5.7 (*SD* 4.29)	.002
Arousal subscale	11.10 (*SD* 3.72)	6.80 (*SD* 4.15)	4.3 (*SD* 3.02)	.001
Anxiety subscale	20.10 (*SD* 6.43)	10.50 (*SD* 8.92)	9.6 (*SD* 9.78)	.013
1 week visual analogue scale	8.60 (*SD* 1.17)	3.50 (*SD* 2.99)	5.1 (*SD* 3.03)	<.001
Visual analogue scale (EQ‐VAS)	50 (*SD* 19.43)	71.60 (*SD* 26.24)	−21.6 (*SD* 27.8)	.036

Note

All measures exhibited a statistically significant improvement 1 week after intervention.

## 4 DISCUSSION

The use of therapies involving motor tasks has been studied previously. One example is *Eye Movement Desensitization and Reprocessing* developed by Shapiro (2014), which consists of eight phases utilizing a specific pattern of ocular movements and sounds (Shapiro, 1989). The use of motor and visuospatial tasks to mitigate intrusive memories was previously tested through an experimental model of PTSD (Holmes et al., 2004). The visuospatial interventions were compared to control conditions such as cognitive or verbal tasks (e.g., quiz, questionnaires, counting backwards). Patients who performed visuospatial tasks had better outcome (Holmes et al., 2010; Iyadurai et al., 2018) suggesting that tasks act not only by distraction but by means of interference with sensory aspects of intrusive memory. (Iyadurai et al., 2018; Kessler et al., 2020).

Although sufferers may believe they can do nothing to ameliorate traumatic memories (Arntz et al., 2005), interference with associated processes may render them amenable to change. Our results may reflect that the tasks are not just simple distractions, but rather compete against the same cognitive resources used to recall the memories, so when two tasks make a demand on attentional capacity the primary task deteriorates (Schubert & Lee, 2009). The structures responsible for the genesis of the trauma may be the same as those that interfere with its modification.

Thus, a task that engages the attentional cluster, as well as working memory (such as the tapping in our therapy) positively interferes with memory processing(Deeprose et al., 2012). This generates a change in the consolidation of memory. (Postman & Underwood, 1973) As seen in other reconsolidation‐based interventions, when the patients engaged on finger tapping, the quality of the memory deteriorates and gets integrated into long‐term memory where the memory becomes less vivid and less emotional (Schubert & Lee, 2009).

Two of our patients reported a slight increase in agitation and anxiety in the 3 days following the therapy and then improved. Other studies have shown similar results. Although the temporary exacerbation of the symptoms might produce discomfort, this malaise should not be considered a contraindication. The benefits of the treatment warrant its usage. (Foa et al., 2002).

The average increase of 21.6 in the EQ‐VAS translates into a direct and real improvement in the patients' quality of life. In this pilot study, all patients reported some benefit, which justifies further investigation. In addition, Motor Interference Therapy is easy to apply, requires few resources, and can be administered in only 30 min.

## 5 CONCLUSION

We present a novel intervention of finger tapping to audio cues with promising results evidenced by statistically significant improvement on all measures 1 week after the study. Motor interference could be a useful tool in the treatment of distressing traumatic memories if these findings can be replicated in a double‐blind controlled study powered to demonstrate efficacy.

## CONFLICT OF INTEREST

None for all the authors.

## AUTHOR CONTRIBUTIONS

Morales‐Rivero Alonso conceived the presented idea and designed the implementation of the research, recruited the sample, performed the intervention collected the data, contributed with data analysis tools, drafted the manuscript, and designed the figures. Reyes‐Santos Lorena conceived the presented idea, designed the implementation of the research, recruited the sample, performed the intervention, collected the data, contributed with data analysis, and drafted the manuscript. Bisanz Erik conceived the presented idea, designed the implementation of the research, and drafted the manuscript. Ruiz‐Chow Angel designed the implementation of the research, performed the intervention, collected the data, and contributed with data analysis. Crail‐Melendez Daniel conceived the presented idea and designed the implementation of the research, recruited the sample, performed the intervention collected the data, contributed with data analysis tools, performed the analysis, supervised the findings of this work, drafted the manuscript, and designed the figures. All authors discussed the results and contributed to the final manuscript.

## ETHICAL APPROVAL

This study received ethical approval from the ethics committee of the National Institute of Neurology and Neurosurgery in accordance with the Declaration of Helsinki.

### Peer Review

The peer review history for this article is available at https://publons.com/publon/10.1002/brb3.1984.

## Data Availability

The data for this study are available from the corresponding author upon reasonable request.
